# Identifying Lanthanide Energy Levels in Semiconductor Nanoparticles Enables Tailored Multicolor Emission through Rational Dopant Combinations

**DOI:** 10.1021/acs.accounts.5c00116

**Published:** 2025-04-11

**Authors:** Gouranga H. Debnath, Prasun Mukherjee, David H. Waldeck

**Affiliations:** † Centre for Nano and Material Sciences, Jain University, Bangalore, Karnataka 562112, India; ‡ Centre for Research in Nanoscience and Nanotechnology, University of Calcutta, Kolkata, West Bengal 700106, India; § Department of Chemistry, University of Pittsburgh, Pittsburgh, Pennsylvania 15260, United States

## Abstract

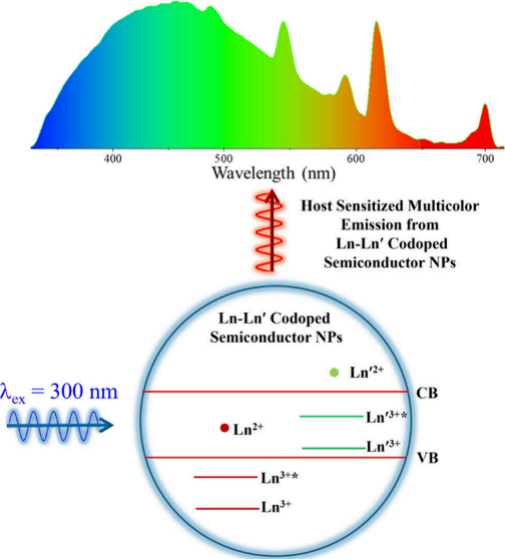

The unique photon emission signatures of trivalent lanthanide cations (Ln^3+^, where Ln = Ce, Pr, Nd, Sm, Eu, Gd, Tb, Dy, Ho, Er, Tm, and Yb) enables multicolor emission from semiconductor nanoparticles (NPs) either through doping multiple Ln^3+^ ions of distinct identities or in combination with other elements for the creation of next-generation light emitting diodes (LEDs), lasers, sensors, imaging probes, and other optoelectronic devices. Although advancements have been made in synthetic strategies to dope Ln^3+^ in semiconductor NPs, the dopant(s) selection criteria have hinged largely on trial-and-error. This combinatorial approach is often guided by treating NP–dopant(s) energy transfer dynamics through the lens of spectral overlap. Over the past decade, however, we have demonstrated that the spectral outcomes correlate better with the placement of Ln^3+^ energy levels with respect to the band edges of the semiconductor, and oxide, host.

In this Account, we describe how the Ln^3+^ energy level alignments affect the dopant emission intensities and dictate interdopant energy transfer processes in semiconductor nanoparticle hosts. This Account begins with a concise primer on the emission characteristics of trivalent lanthanides, the challenges that are associated with realizing meaningful lanthanide luminescence, and how semiconductor nanoparticles can act as a host to sensitize lanthanide emission. We then describe a semiempirical approach that can be used to place the lanthanide ground and luminescent energy levels with respect to the band edges of the host semiconductor nanoparticle. The ability of this model to track and predict the lanthanide sensitization efficiency is illustrated for singly doped zinc sulfide (ZnS), titanium dioxide (TiO_2_), and cesium lead chloride (CsPbCl_3_) perovskite hosts. Next, we discuss how knowledge of energy level offsets can be used to select dopant(s) for tunable multicolor emission by identifying different charge trapping processes for semiconductors doped with single and multiple lanthanides and discussing their impact on sensitization outcomes. Following this discussion, the Account lists viable Ln^3+^ combinations in ZnS NPs based on the charge trapping model and shows the limitations of spectral overlap models in predicting viable Ln^3+^ dopant combinations. Feasible f-f and d-f codopant combinations based on charge trapping are presented for TiO_2_ and CsPbCl_3_ NPs. The intricacies of interdopant energy migration and spin considerations that dictate the dopant(s) sensitization efficiencies are made known. Finally, we use these considerations to predict NP–dopant(s) combinations that should exhibit concerted emissions from the blue to the near-infrared (NIR) region, thereby enabling the design of bespoke optoelectronic properties. The Account ends with some forward-looking thoughts, arguing for the need to develop better quantitative models in order to explore the Ln^3+^ sensitization mechanisms and presenting ideas for applications of doped semiconductor NPs in energy and health that would be aided by interdopant energy transfer dynamics.

## Key References

- MukherjeeP.; ShadeC. M.; YinglingA. M.; LamontD. N.; WaldeckD. H.; PetoudS.Lanthanide Sensitization in II–VI Semiconductor Materials: A Case Study with Terbium(III) and Europium(III) in Zinc Sulfide Nanoparticles. J. Phys. Chem. A**2011**, 115, 4031–404121090795 10.1021/jp109786wPMC3061249.^1^*This is the first report to interpret Ln*^*3+*^*emission efficiences in Ln*^*3+*^-*doped II–VI sulfide and selenide semiconductors by charge trapping mediated sensitization mechanisms through informed placements of Ln*^*3+*^*energy levels relative to the semiconductor nanoparticles’ band edges*.
- ChakrabortyA.; DebnathG. H.; SahaN. R.; ChattopadhyayD.; WaldeckD. H.; MukherjeeP.Identifying the Correct Host–Guest Combination to Sensitize Trivalent Lanthanide (Guest) Luminescence: Titanium Dioxide Nanoparticles as a Model Host System. J. Phys. Chem. C**2016**, 120, 23870–23882.^2^*This is the first defining report where the trends of Ln*^*3+*^*emissions in titania (TiO*_*2*_*) nanoparticles (as a model oxide semiconductor) were explained using Ln*^*3+*^*-TiO*_*2*_*energy alignment principles*.
- DebnathG. H.; BloomB. P.; TanS.; WaldeckD. H.Room Temperature Doping of Ln^3+^ in Perovskite Nanoparticles: A Halide Exchange Mediated Cation Exchange Approach. Nanoscale**2022**, 14, 6037–605135383344 10.1039/d2nr00490a.^3^*This is the first work that rationalizes Ln*^*3+*^*emissions in cesium lead chloride (CsPbCl*_*3*_*) perovskite nanoparticles by positioning Ln*^*3+*^*energy levels relative to the band edges of CsPbCl*_*3*_*nanoparticles*.

## Introduction

The f-orbital transitions of trivalent lanthanide cations (Ln^3+^, where Ln = Ce, Pr, Nd, Sm, Eu, Gd, Tb, Dy, Ho, Er, Tm, and Yb) endow them with sharp emission band signatures that span the ultraviolet (UV) (Gd^3+^), entire visible (Pr^3+^, Sm^3+^, Eu^3+^, Tb^3+^, Ho^3+^, Er^3+^, Tm^3+^), and near-infrared (NIR) (Nd^3+^, Er^3+^, Tm^3+^, Yb^3+^) spectral window.^4−7^ The spectral positions of Ln^3+^ ions remain insensitive to perturbations in their local microenvironment, temperature, or pH; and they exhibit microsecond to millisecond emission lifetimes, due to the parity forbidden nature of the optical transitions. In addition, they are highly resistant to photobleaching.^4−7^ For optical pumping applications, the poor optical absorptivity of the Ln^3+^ 4f-4f transitions, which arise from the Laporte selection rules,^8^ can be circumvented by the antenna effect, i.e., pumping the high absorptivity semiconductor NP transitions, or 4f-5d Ln^3+^ transitions, and funneling the energy into accepting energy levels of Ln^3+^ to generate excited states of Ln^3+^*.^9,10^ The semiconductor NP host can also inhibit the quenching of Ln^3+^* emission by vibrational modes of solvents and ligands.^11,12^ Doping semiconductor nanoparticles (NPs) with multiple Ln^3+^ species or in combination with luminescent d-block elements has been shown to produce multicolor emission by triggering concerted emissions from both the semiconductor (exciton) and the dopant(s) centers. This approach promises to revolutionize the development of next generation light emitting diodes (LEDs), lasers, sensors, imaging probes, photovoltaics, telecommunications, and other optoelectronic devices.^13−21^

The selection of optimal d-f or f-f dopant pairs for a particular host semiconductor NP and an understanding of the underlying interdopant and NP-dopant(s) electronic interactions are essential for tailoring applications in multiplexing. Early reports on Ln^3+^ emissions in singly doped semiconductor NPs attributed the sensitization of the Ln^3+^ emissions to energy transfer mechanisms that rely on semiconductor NP-Ln^3+^ donor–acceptor spectral overlap.^22−24^ This mechanism was not borne out, however, and workers reverted to a trial-and-error approach for selecting combinations of host semiconductor NPs and Ln^3+^ ions.^18,25^ Since 2011, our sustained efforts on studying single and codoped Ln^3+^ in a series of II–VI sulfides and selenides,^1,26−28^ IV–VI oxides,^2,29−31^ and metal halide perovskite^3,32^ semiconductor NPs have revealed that the positioning of the Ln^3+^ energy levels with respect to the band edges of the host semiconductor can be used to rationalize the sensitization efficiencies of Ln^3+^ in semiconductor NPs. Below we discuss how the energy level alignment of the Ln^3+^ electronic states to that of the semiconductor host provides an efficient guide to predict viable f-f or d-f combinations with a high consistency between predicted and observed outcomes. This account emphasizes the ability to move beyond traditional trial-and-error approaches and advocates design principles to predict possible NP-dopant(s) combinations with bespoke spectral properties, such as concerted emissions from the blue to the near-infrared (NIR).

## Rules to Generate a Semiempirical Energy Level Scheme

Our research has exploited the predictable differences in electronic configuration energies of lanthanide ions to probe how the energy offset between the lanthanide ions and a semiconductor host’s band edges affect luminescence sensitization. The ground state electron configurations of the lanthanide ions Ln^2+^ and Ln^3+^ are [Xe]4f^n^ and [Xe]4f^n-1^, respectively; and a plot of their ionization energy versus atomic number show a zigzag shape with the most stable configurations appearing at half-filled and filled f-shells. More than this, the f-orbital electron density is well shielded from interactions with neighboring atoms so that ligand field (and crystal field) effects on the electron energetics is weak.^33^ These facts mean that the variation of the lanthanide dopant’s identity can be used to systematically probe how the energy level position of the dopant ion relative to the band edge of a semiconductor host nanoparticle affects luminescence sensitization. Note, however, that the absolute energy level position of the lanthanide ions in a host can vary considerably with the anion identity of a semiconductor, and it is necessary to find this energy offset for proper placement of the lanthanide series energy positions.

**Figure 1:**
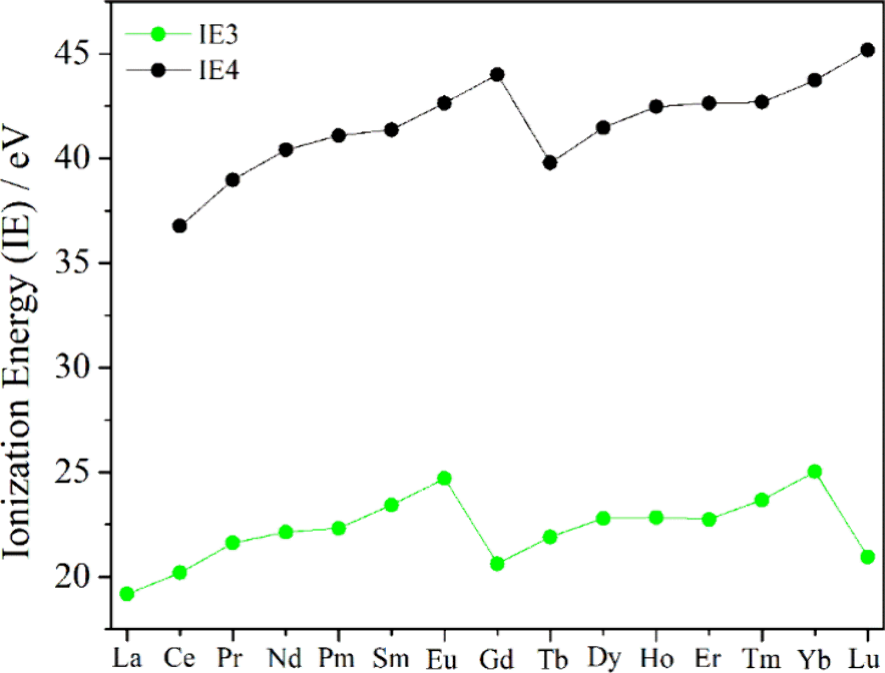
Third (green) and fourth (black) gas phase ionization energies (IEs) of lanthanides are shown. These values are taken from the reports of Sugar and co-workers.^34^

Building on earlier work by Dorenbos and by Jørgensen, we use the following assumptions to place the ground state energy levels for Ln^3+^ f-orbital states in a semiconductor NP:^33−38^

i. The core-like nature of the Ln^3+^ leads to a universal trend in their electron orbital energies that is independent of the host semiconductor. This can be visualized from the third and fourth ionization energies (IE) of lanthanides as shown in Figure 1. Additionally, the ground state energy of Ln^3+^ (*E*_*Ln*3+_) and Ln^2+^ (*E*_*Ln*2+_) can be estimated from their gas phase ionization energies, i.e. IE4 and IE3 respectively, according to the eq 1:^35^
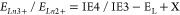
1where E_L_ is a constant energy shift, related to the Madelung potential of the host matrix, for all lanthanides, and X is a correction factor associated with the lanthanide contraction, i.e., it accounts for changes in Madelung potential and lattice relaxation that arise from the Ln ion size change. Relevant *E*_*Ln*3+_/*E*_*Ln*2+_ values are reported in ref (35).
ii. The charge transfer energy (E_CT_) from the anion of the host semiconductor to an Eu^3+^ dopant is equal to the energy difference between the top of the valence band and the Eu^2+^ ion’s ground energy level. That is, the energy to move an electron from the anion of the semiconductor host to Eu^3+^, generating an Eu^2+^ dopant ion, is assumed to be equal to the energy between the Eu^2+^ dopant ion ground state and the valence band edge of the semiconductor. This assumption is often reasonable because the anion orbitals comprise the valence band edge of many common semiconductors.
iii. The energy difference between the Eu^3+^ and the Eu^2+^ ground energy level in the semiconductor host is much smaller than that for the gas phase, and we assume that it is 5.7 eV for semiconductor host materials with a band gap <6 eV.(37)

With these assumptions, it is only necessary to find the E_CT_ and scale the *E*_*Ln*__3+_/*E*_*Ln*__2+_ based on the E_CT_ to construct the energy diagram. Although it is best to determine E_CT_ experimentally, it is not always possible. In these cases, it can be estimated from Pauling’s electronegativity (η) scale and Jørgensen’s relationship^35,37,39^ according to the eq 2:
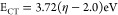
2

Following the positioning of Ln ground states, the placement of the top of the valence band (VB) at zero helps position the bottom of the conduction band (CB) according to the band gap of the NP and has led to the successful interpretation of the observed Ln^3+^ emission trends in doped semiconductor NPs. For visualizing charge trapping/detrapping processes in a given host, such relative energy level positions serve the purpose.

**Figure 2:**
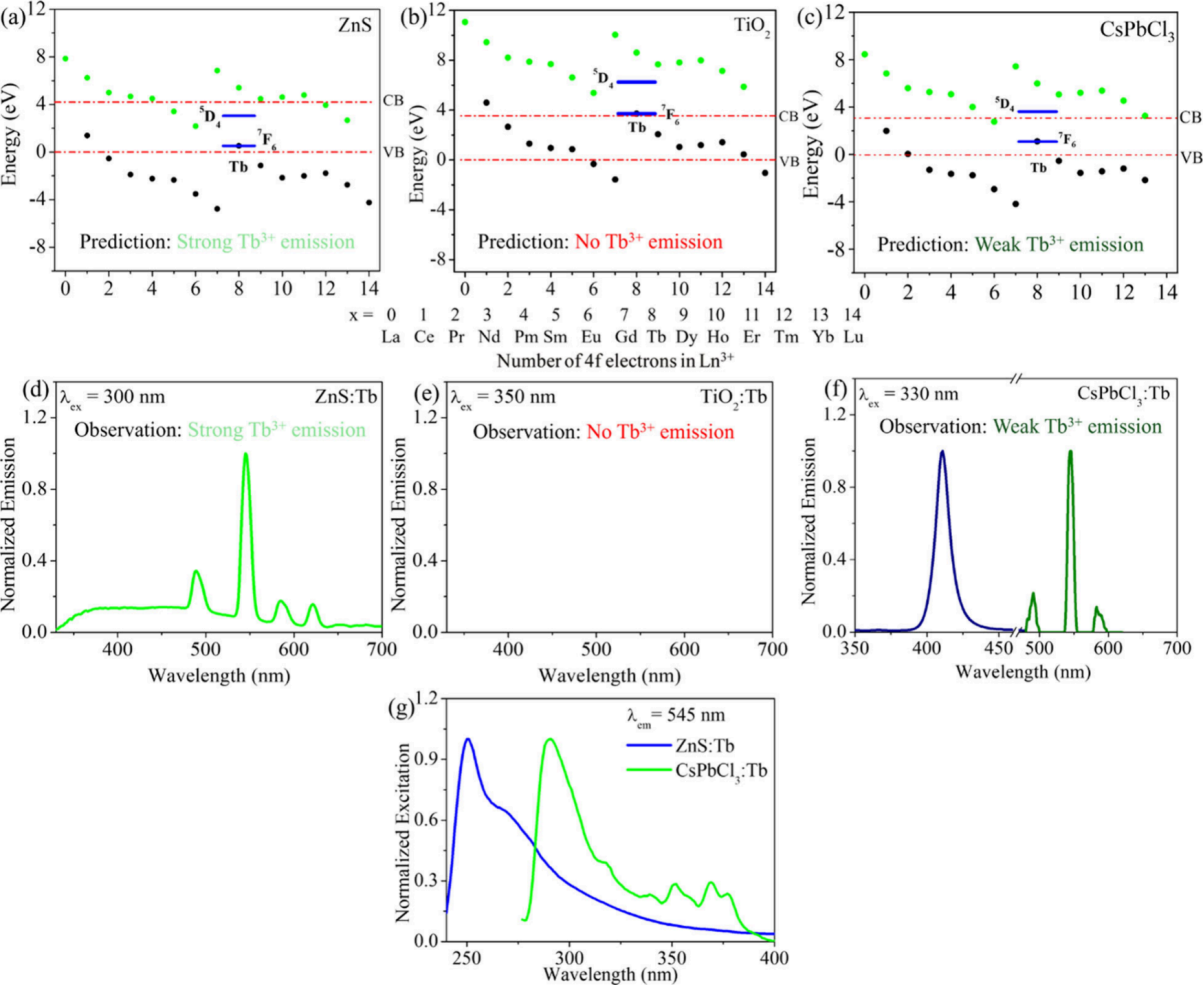
Panels (a–c) show the location of the Ln^3+^ (black dots) and Ln^2+^ (green dots) ground states in ZnS (average NP diameter of 3.3 ± 0.4 nm), TiO_2_ (average diameter of 3.5 ± 0.4 nm), and CsPbCl_3_ (average edge length of 8.4 ± 0.9 nm) NPs with corresponding band gaps of 4.20, 3.54, and 3.15 eV, respectively. The NP valence band (VB) edge is set to zero in each case. The Tb^3+^ ground (^7^F_6_) and luminescent (^5^D_4_) energy levels are presented in blue. Panel (d) shows the steady-state emission spectra in Tb^3+^-doped ZnS NPs with the broad ZnS centered emission in the 320–700 nm region and sharp emission bands centered at 490, 545, 585, and 620 nm originating from Tb^3+^^5^D_4_ → ^7^F_*J*_ transitions (where *J* = 6, 5, 4, 3). Following optical excitation, the Tb^3+^^5^D_4_ and ^7^F_6_ levels in ZnS are optimally positioned to trap electron–hole pairs, and their eventual recombination generates a Tb^3+^* excited state and its radiative emission. Tb^3+^ emission was not observed in doped TiO_2_ NPs as shown in panel (e), as the Tb^3+^ levels are placed above the conduction band (CB) and are unable to trap electron–hole pairs. Panel (f) shows the steady-state emission profile of Tb^3+^-doped CsPbCl_3_ NPs, where the perovskite-centered emission (navy) at 410 nm is accompanied by three moderate-weak Tb^3+^ emission bands (olive) at 490, 545, and 585 nm. The emission here is weaker than that of ZnS and is attributed to the placement of the ^5^D_4_ level above the CB, which allows autoionization to compete with charge trapping. Panel (g) shows the normalized excitation spectra of ZnS:Tb (adapted with permission from ref (26), copyright 2015 American Chemical Society) and CsPbCl_3_:Tb NPs generated by monitoring the Tb^3+^ emission band at 545 nm. Panels (a) and (d) are adapted with permission from ref (1). Copyright 2011 American Chemical Society and constructed based on the information reported. Panel (b) is adapted with permission from ref (2). Copyright 2016 American Chemical Society. Panels (c) and (f) are adapted with permission from ref (3). Copyright 2024 Royal Society of Chemistry.

The data in Figure 2 for the sensitization of Tb^3+^* illustrates the effectiveness of this energy level scheme for predicting sensitization. Figure 2 shows the placement of Ln^3+^ and Ln^2+^ ground states in NPs of the II–VI sulfide ZnS (panel a);^1^ the IV–VI oxide TiO_2_ (panel b),^2^ and the metal halide perovskite CsPbCl_3_ (panel c);^3^ and it tracks the Tb^3+^ (the brightest among the Ln^3+^ that emit in the visible spectral region, based on environmental quenching effects) ground (^7^F_6_) and luminescent (^5^D_4_) energy levels across these systems. From the energy diagrams and photophysical relaxation considerations, we expect the Tb^3+^* to be most populated for ZnS, somewhat less populated for CsPbCl_3_, and least populated for TiO_2_. In fact, we observe a Tb^3+^ emission quantum yield of 5% in ZnS, no detectable emission in TiO_2_, and 0.15% in CsPbCl_3_.^1,3^ A comparison of the excitation spectra in ZnS and CsPbCl_3_ (panel g) generated by monitoring the Tb^3+^ emission band at 545 nm shows a higher contribution of Tb^3+^ direct excitation bands in the 350–400 nm region for CsPbCl_3_, substantiating that ZnS is the better sensitizer. The agreement between the Tb^3+^* emission spectra (panels (d) through (f)) and the expected population of the ^5^D_4_ state of Tb^3+^* is remarkable. The intricacies of synthetic conditions, Ln^3+^ doping strategies, ligand shell effects, placement of distinct Ln^3+^ energy levels, along with band edge or spectral overlap considerations and charge trapping pathways for a range of Ln^3+^ doped semiconductor NPs, are compiled and discussed in our recent review articles.^9,10,40^

## Charge Trapping Scenarios in Single and Co-Doped Semiconductor NPs

**Scheme 1:**
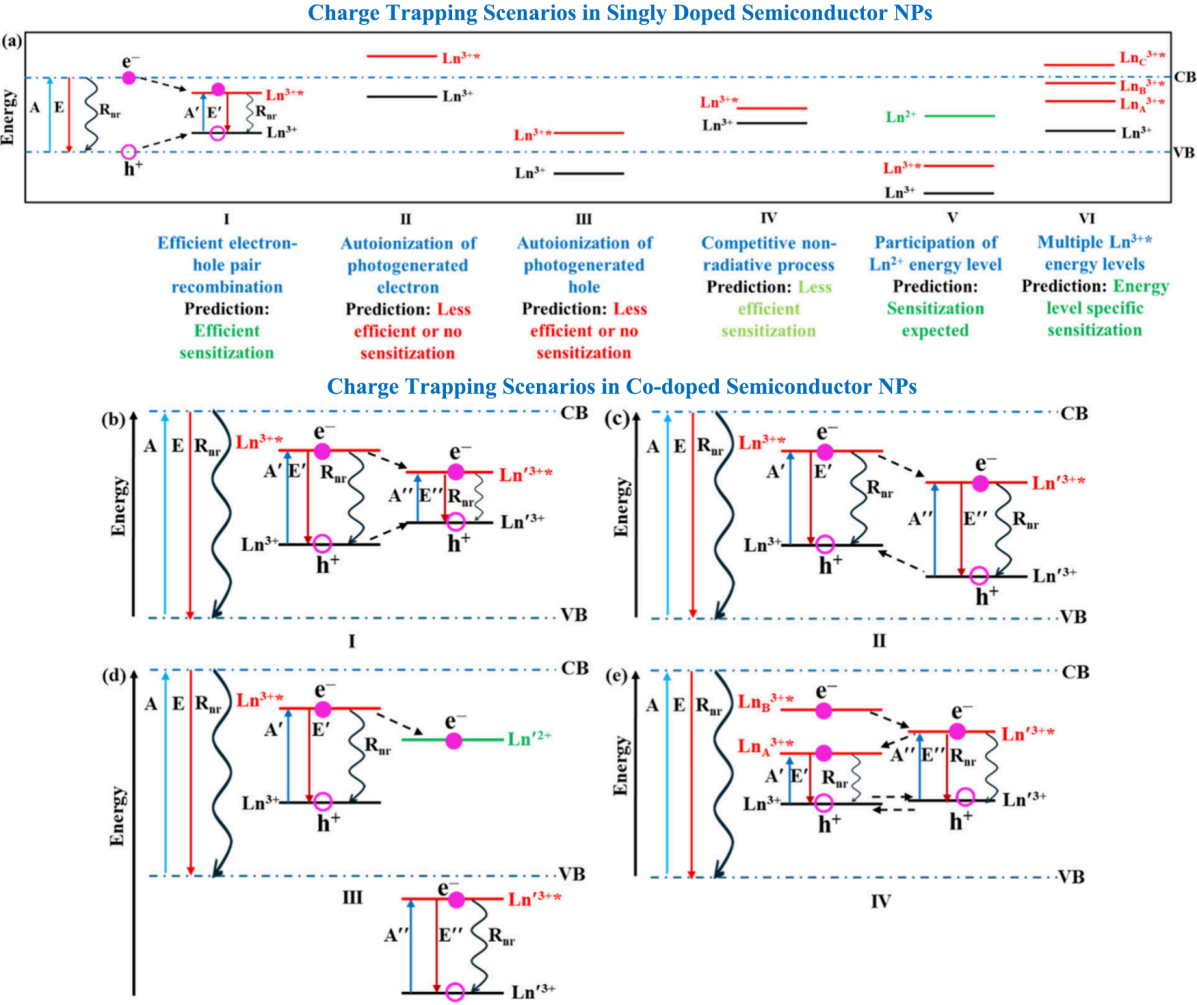
Charge trapping scenarios in Ln^3+^ single and co-doped semiconductor NPs are shown in panels (a) and (b–e), respectively. A and E represent the NP band edge absorption and emission. A′(A″) and E′(E′′) represent the Ln^3+^ (Ln′^3+^) absorption and emission. R_nr_ represents nonradiative relaxation processes. The dashed arrows indicate electron and hole migration pathways.

Scheme 1 shows some charge trapping scenarios in Ln^3+^ single (panel a) and codoped (panels b-e) semiconductors and discusses the Ln^3+^ sensitization outcomes. The energy alignments in Case I of panel (a) depict an ideal scenario where the optimal placement of the Ln^3+^* and Ln^3+^ levels lead to efficient capture and recombination of electron–hole pairs, displaying efficient sensitization. Cases II and III are predicted to have less efficient or no sensitization because of autoionization processes; the Ln^3+^* level in case II is located above the conduction band (CB) and the Ln^3+^ level in case III is located below the valence band. The closely spaced Ln^3+^* and Ln^3+^ levels in case IV are predicted to display less efficient sensitization as nonradiative processes like phonon emission can compete with radiative emission from excited Ln^3+^*. In addition, the significant energy difference between the band edges and the Ln^3+^ levels leads to less efficient electron–hole pair colocalization at the dopant site, making them less competitive with other nonradiative decay pathways. Case V shows a scenario where the charge trapping involving the Ln^2+^ ground state can result in the population of excited Ln^3+^* and eventual sensitization. For example the Eu^3+^ sensitization mechanism in doped ZnS NPs involves the Eu^2+^ ground state and falls in this category.^9^ Case VI shows a lanthanide with multiple Ln^3+^* (Ln_A_^3+^*, Ln_B_^3+^*, Ln_C_^3+^*) levels and predicts specific emission scenarios from these Ln^3+^* based on their placement. For example, emission from Ln_C_^3+^* is unlikely as it is placed above the CB. Emission from Ln_B_^3+^* should be weak as autoionization can compete with charge trapping while the emission from Ln_A_^3+^* should be efficient.

Panels b-e of Scheme 1 show four types of charge trapping possibilities for Ln-Ln′ co-doped semiconductor NPs. In type I (panel b), the energy level alignment can result in the migration of a trapped electron and hole from Ln to Ln′; while in the type II energy alignment (panel c) only the migration of a trapped electron from Ln to Ln′ is favored. Panel d, which we call type III energy alignment, shows the Ln′^3+^* and Ln′^3+^ levels placed below the valence band (VB), and energy migration from Ln to Ln′ is facilitated by the Ln′^2+^ which can accept electrons from Ln^3+^*. Panel e, which we call Type IV energy alignment, shows the interaction between Ln with multiple luminescent energy levels and Ln′ with a single luminescent energy level. Emission from Ln_A_^3+^* is facilitated by electron migration from Ln_B_^3+^* to Ln′^3+^* and then from Ln′^3+^* to Ln_A_^3+^*. The realization of both Ln and Ln′ emissions from types I–IV is possible but with varying intensities.

## Viable Ln^3+^ Co-dopant Combinations in ZnS NPs

**Figure 3:**
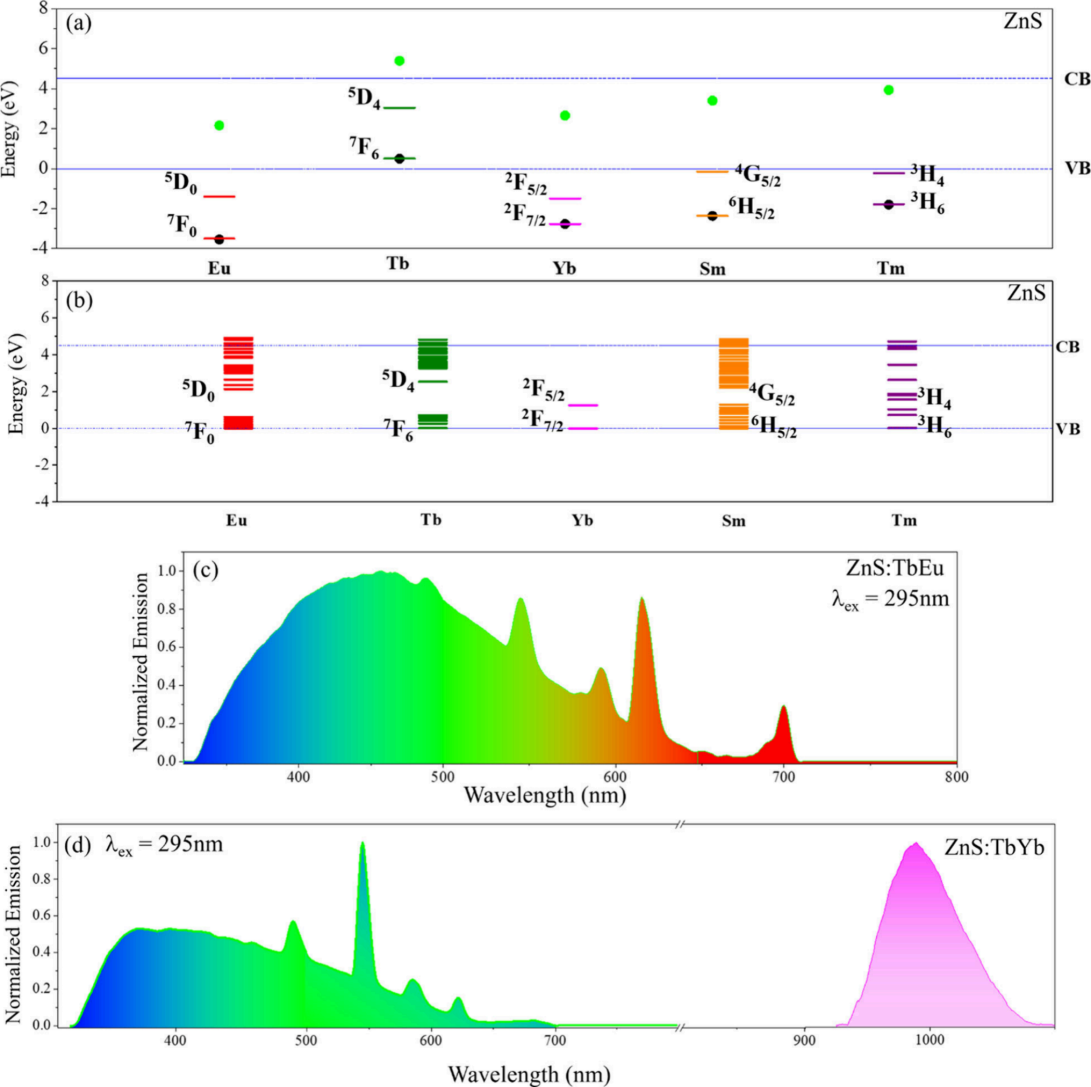
Panel (a) shows the placement of the ground and luminescent energy levels of Eu^3+^(^7^F_0_ and ^5^D_0_), Tb^3+^(^7^F_6_ and ^5^D_4_), Yb^3+^(^2^F_7/2_ and ^2^F_5/2_), Sm^3+^(^6^H_5/2_ and ^4^G_5/2_), and Tm^3+^(^3^H_6_ and ^3^H_4_) in ZnS NPs (band gap = 4.2 eV) according to the charge trapping model. The Ln^3+^ and Ln^2+^ ground states are indicated by black and green dots, respectively. Panel (b) shows the Eu^3+^, Tb^3+^, Yb^3+^, Sm^3+^, and Tm^3+^ energy levels in the ZnS NP according to the spectral overlap model. For constructing the energy levels in panel (b), both the valence band (VB) of the NPs and the respective Ln^3+^ ground energy levels are placed at 0 eV, and the Ln^3+^ higher-lying energy levels are placed accordingly. The steady-state emission spectra of ZnS NPs co-doped with Tb^3+^−Eu^3+^ and Tb^3+^−Yb^3+^ pairs are shown in panels (c) and (d) respectively. Adapted with permission from ref (28). Copyright 2022 American Chemical Society.

Following the tests of charge trapping and the predictions of Tb^3+^ emission efficiency in singly doped ZnS, TiO_2_, and CsPbCl_3_ NPs, we now discuss how the energy level scheme helps identify viable Tb^3+^−Ln′^3+^ co-dopant pairs in ZnS NPs.^28^Figure 3(a) shows the ground and luminescent energy levels of Eu^3+^, Tb^3+^, Yb^3+^, Sm^3+^, and Tm^3+^ in ZnS NPs. The important parameter to consider is ΔE[Tb^3+^*−Ln′^2+^], the energy difference (ΔE) between the Tb^3+^ luminescent state (LS) and the Ln^2+^ ground state (GS) of the co-dopants Eu, Yb, Sm, and Tm (see Table 1). A positive ΔE[Tb^3+^*−Ln′^2+^] indicates favorable alignment of Tb^3+^−Eu^3+^ and Tb^3+^−Yb^3+^ energy levels via the Eu^2+^ and Yb^2+^ ground states. For example, following optical excitation and charge trapping at the Tb^3+^ center, the electrons from the ^5^D_4_ Tb^3+^* level can migrate using the lower lying Eu^2+^ or Yb^2+^ levels. Recombination of electrons from the Eu^2+^ or Yb^2+^ levels with holes from the valence band or from Tb^3+^^7^F_6_ can populate the excited Eu^3+^* and Yb^3+^* resulting in Eu^3+^ or Yb^3+^ emissions in the presence of Tb^3+^. Conversely, the negative ΔE[Tb^3+^*−Ln′^2+^] value for Tb^3+^−Sm^3+^ and Tb^3+^−Tm^3+^ indicate that electron migration from the ^5^D_4_ Tb^3+^* level to Sm^2+^ and Tm^2+^ is not viable. These considerations are confirmed by the steady-state emission spectra in Figures 3(c) and 3(d). The spectrum for Tb^3+^−Eu^3+^ co-doped ZnS NPs (Figure 3(c)) shows a broad ZnS NP emission centered near 400 nm and the characteristic Tb^3+^ and Eu^3+^ emission bands at 490, 545, 590, 616, and 700 nm. Figure 3(d) shows the spectrum for Tb^3+^−Yb^3+^ codoped ZnS NPs in which the ZnS and Tb^3+^ emission bands appear with the Yb^3+^ emission centered at 980 nm corresponding to its ^2^F_5/2_→^2^F_7/2_ transition. Sm^3+^ and Tm^3+^ emissions in Tb^3+^−Sm^3+^ and Tb^3+^−Tm^3+^ co-doped cases were not observed. In contrast, predictions based on the spectral overlap model, which can be deduced from the diagram for the ZnS bandgap and the Ln^3+^ energy gaps shown in Figure 3(b), erroneously imply that Tb^3+^−Sm^3+^ and Tb^3+^−Tm^3+^ pairs as viable co-dopants in ZnS, a false positive (see Table 1). In earlier work, we showed that the computed overlap integrals considering both the Förster and Dexter energy transfer mechanisms in single Ln^3+^ doped II–VI sulfide and selenide semiconductor NPs are unable to rationalize the host sensitized Ln^3+^ emission.^10^

**Table 1:** Comparison of Experiment with the Charge Trapping and Spectral Overlap Model Predictions for Inter-Dopant Sensitization between Tb^3+^ and Other Lanthanides in ZnS NPs^a^

	Charge Trapping Mechanism		Spectral Overlap Mechanism		
Tb-Ln′	ΔE[Tb^3+^* – Ln′^2+^] (eV)	Prediction on Tb^3+^– Ln′^3+^ Electronic Interaction	ΔE[Tb^3+^* – Ln′^3+^ (Nearest Lower Lying Energy Level)] (eV)	Prediction on Tb^3+^– Ln′^3+^ Electronic Interaction	Experimental Observation
Tb-Eu	0.88	Feasible	0.18	Feasible	Observed
Tb-Yb	0.39	Feasible	1.27	Not Feasible	Observed
Tb-Sm	–0.36	Not feasible	0.07	Feasible	Not observed
Tb-Tm	–0.89	Not feasible	0.67	Feasible	Not observed

a Reproduced with permission from ref (28). Copyright 2022 American Chemical Society.

*The charge trapping model can be extended to predict sensitization in other codoped sulfide semiconductors (or size dependent band edge shifts) by adjusting the CB energy level position to account for the changes in the host’s band gap*.

## Viable Ln^3+^ Co-Dopant Combinations in TiO_2_ NPs

**Figure 4:**
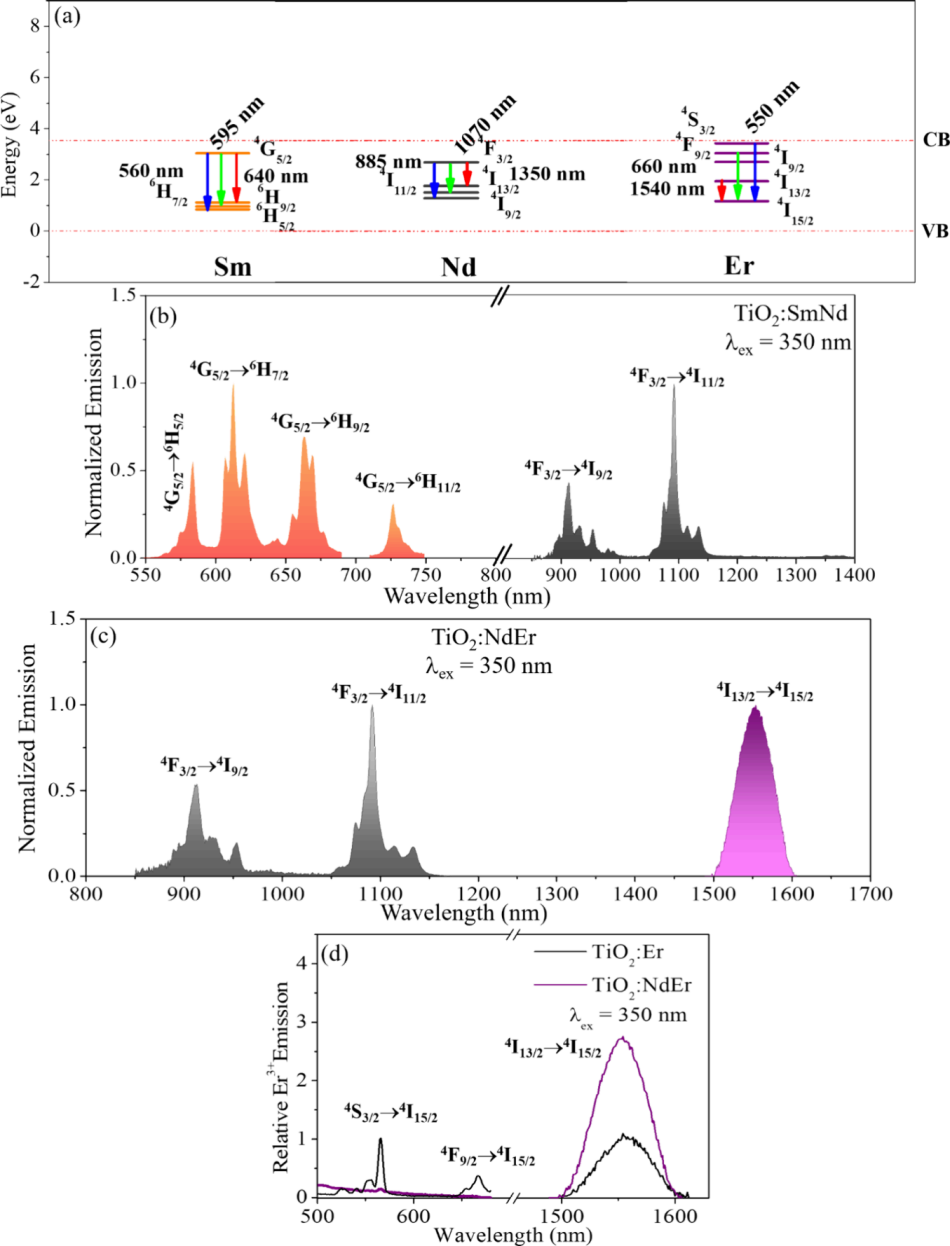
Panel (a) shows the placement of Sm^3+^, Nd^3+^, and Er^3+^ ground and luminescent energy levels in TiO_2_ NPs with a band gap of 3.54 eV. The wavelengths are labeled according to reported values for Ln^3+^–water complexes. The steady-state emission spectra of Sm^3+^−Nd^3+^ and Nd^3+^−Er^3+^ co-doped TiO_2_ NPs are shown in panels (b) and (c) respectively. The Nd^3+^ and Sm^3+^ emission quantum yields in the TiO_2_:NdSm NPs (panel b) are 1.5% and 0.53% respectively. The Nd^3+^ and Er^3+^ NIR emission quantum yields in the TiO_2_:NdEr NPs (panel c) are 1.7% and 0.00016%, respectively. Panel (d) compares the relative intensities of Er^3+^ in singly doped TiO_2_ NPs versus Nd^3+^−Er^3+^ codoped TiO_2_ NPs. Panel (a) is adapted with permission from ref (2). Copyright 2016 American Chemical Society. Panels (b) and (d) are adapted with permission from ref (29). Available under a CC-BY-NC license. Copyright 2017 Royal Society of Chemistry/the authors. Panel (c) is adapted from ref (30). Copyright 2018 with permission from Elsevier.

The predictions of the energy level scheme for TiO_2_ NP hosts, which display sensitization for singly doped Nd^3+^, Sm^3+^, Eu^3+^, Ho^3+^, Er^3+^, Tm^3+^, and Yb^3+^ and no sensitization for Pr^3+^, Gd^3+^, Tb^3+^, and Dy^3+^ dopants, are in excellent agreement with experiment; see ref.^2^Figure 4a shows ground and luminescent energy levels of Sm^3+^, Nd^3+^, and Er^3+^ in TiO_2_ NPs. The co-dopant pair Sm^3+^−Nd^3+^ leads to six concerted emission bands, ranging from the visible to the NIR, with Sm^3+^ emissions centered at 584, 612, 664, and 726 nm and Nd^3+^ emissions centered at 912 and 1094 nm [see Figure 4(a), (b)].^29,30^ Similarly, the Nd^3+^−Er^3+^ pair in TiO_2_ NPs displays concerted emission bands in the NIR I and NIR II regions (see Figure 4c) with typical Nd^3+^ emission bands along with the Er^3+^ emission at 1550 nm.^29,30^ Note that the placement of the Nd^3+^^4^F_3/2_ luminescent energy level below the Er^3+^^4^S_3/2_ and ^4^F_9/2_ luminescent levels in Figure 4a predicts favorable electron migration from the Er^3+^ levels to Nd^3+^ levels. This expectation is validated by the nearly flat Er^3+^ emission signatures in the visible region for co-doped TiO_2_:NdEr NPs when compared to singly doped TiO_2_:Er NPs (see Figure 4d). Figure 4(a) also predicts that the Nd^3+^^4^F_3/2_ luminescent energy level can act as a funnel to populate the Er^3+^^4^I_13/2_ luminescent energy level. Also, the Nd^3+^^4^I_9/2_ ground energy level is nearly isoenergetic with the Er^3+^^4^I_15/2_ ground energy level and should accommodate inter-lanthanide hole transfer. Note that, energy transfer from Nd^3+^ to Er^3+^ levels is spin allowed, since ΔS = 0 in these energy levels. Together, these factors boost the Er^3+^ NIR emission by ∼ 3 times in TiO_2_:NdEr NPs when compared to TiO_2_:Er NPs; see Figure 4(d)^29^ The Sm^3+^−Nd^3+^ and Nd^3+^−Er^3+^ emissions in TiO_2_ NPs can be used as noncytotoxic, photobleaching resistant, bioimaging probes^41^ that provide multiplexed imaging capabilities from red to NIR II thereby expanding the library of NIR imaging agents.

*The emission efficiencies of these Ln*^*3+*^*co-dopant pairs can be rationalized, and predicted, for other semiconductor oxide NPs by adjusting the conduction band edge to account for the changes in the band gap energy*.

## Viable Co-Dopant Combinations in CsPbCl_3_ NPs

For singly doped CsPbX_3_ NPs [X = Cl, Br], the d-block dopant Mn^2+^ has been extensively studied because of the emission color tunability it provides based on synthetic conditions, dopant concentration, and halide identity.^42−45^ In a recent computational study, De Angelis and co-workers^46^ estimate the Mn^2+^ ground (^6^A_1_) and luminescent (^4^T_1_) energy levels in CsPbCl_3_ NPs and explain the Mn^2+^ emission by invoking a charge trapping mechanism to circumvent spin and orbital restrictions. Equally well explored are Yb^3+^ doped CsPbX_3_ NPs because of their quantum cutting effects.^47,48^ These studies and our 2022 report on Ln^3+^ [Ln = Nd, Sm, Eu, Tb, Dy, and Yb] doped CsPbCl_3_ emission trends allows us to identify viable d-f and f-f co-dopant pairs for generating multicolor emission.^3,32^

**Figure 5:**
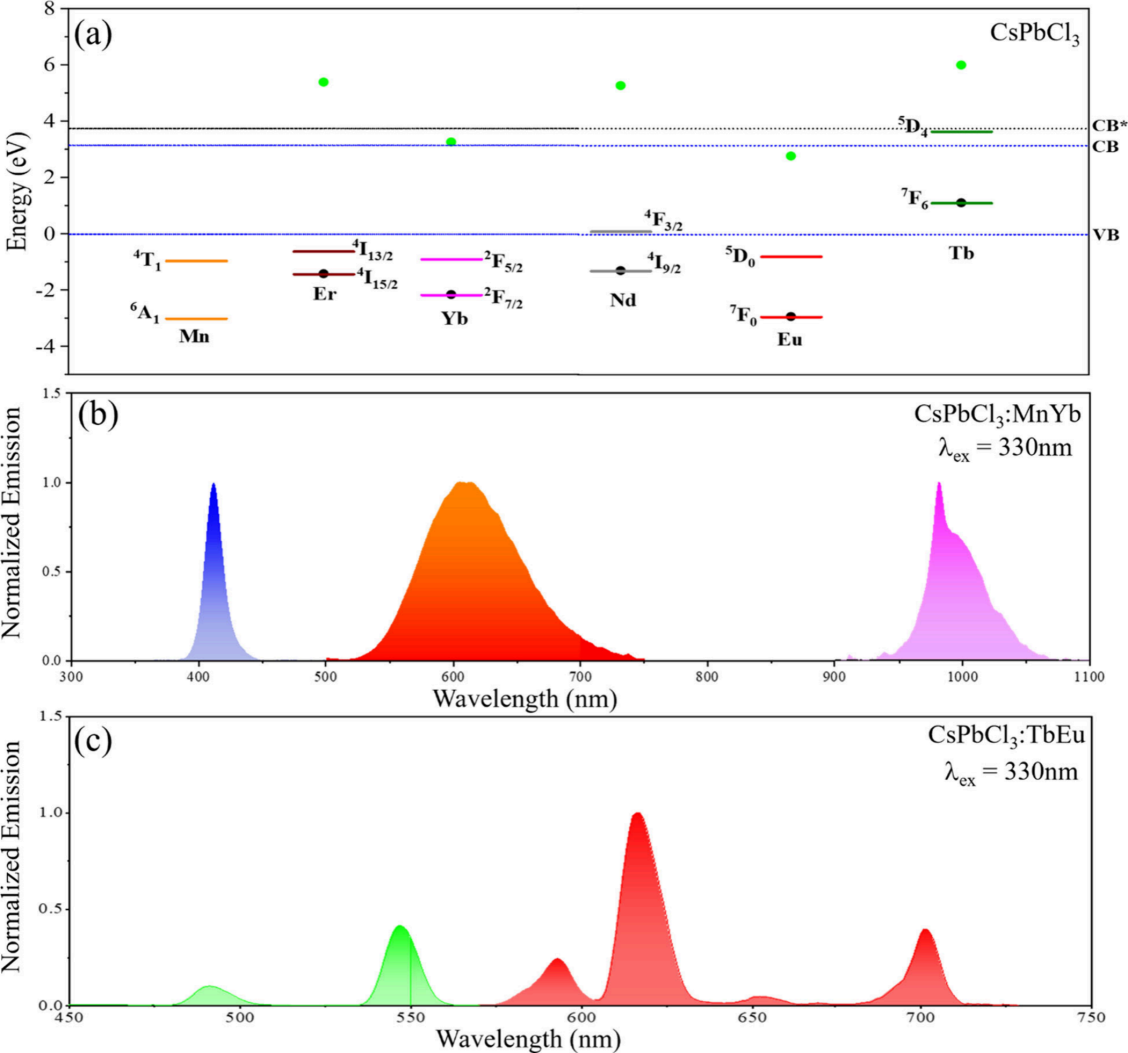
Panel (a) shows the respective ground and luminescent energy levels of Mn^2+^(^6^A_1_ and ^4^T_1_), Er^3+^(^4^I_15/2_ and ^4^I_13/2_), Yb^3+^(^2^F_7/2_ and ^2^F_5/2_), Nd^3+^(^4^I_9/2_ and ^4^F_3/2_), Eu^3+^(^7^F_0_ and ^5^D_0_), and Tb^3+^ (^7^F_6_ and ^5^D_4_) in CsPbCl_3_ NPs (band gap = 3.15 eV) according to the charge trapping model. To construct this energy diagram, an average electronegativity of the Cl and Pb was used to estimate E_CT_ (2.77 eV) because the valence band in CsPbCl_3_ has contributions from the Cl 3p and Pb 6s orbitals.^51^ Note that the other luminescent energy levels of Er^3+^ have not been included for clarity. The Ln^3+^ and Ln^2+^ ground states are indicated by black and green dots, respectively. The steady-state emission spectra of CsPbCl_3_ NPs co-doped with Mn^2+^−Yb^3+^ are shown in panel (b). The CsPbCl_3_, Mn^2+^, and Yb^3+^ emission quantum yields are 1.6%, 6.2%, and 1.0%, respectively. Time-gated emission spectra of CsPbCl_3_ NPs co-doped with Tb^3+^−Eu^3+^ are shown in panel (c). The time-gated modality removes the nanosecond lived components, and a gate time allows the collection of microsecond to millisecond lived species. This is particularly useful in visualizing the Ln^3+^ emission bands in panel (c) because the intense perovskite emission in steady-state masks the Ln^3+^ emissions, a consequence of the difference in the radiative rates of CsPbCl_3_ NPs (nanoseconds) and Ln^3+^ (microseconds-milliseconds). The presence of a higher energy absorption band at 300–310 nm in CsPbCl_3_:TbEu NPs, which correlates with a higher energy perovskite excited state, with contributions from Tb^3+^ 4f–5d energy transitions is shown as CB* in panel (a) and makes the ^5^D_4_ Tb^3+^ level a moderate-weak electron trap. Adapted with permission from ref (3). Copyright 2022 Royal Society of Chemistry and ref (32). Copyright 2024 Royal Society of Chemistry.

Figure 5(a) shows the energy level scheme for some doped CsPbCl_3_ NPs. Note that the ^4^T_1_ level in Mn^2+^ and the ^2^F_5/2_ level of Yb^3+^ in CsPbCl_3_ NPs are positioned to act as electron traps, whereas the ^6^A_1_ level of Mn^2+^ and ^2^F_7/2_ levels of Yb^3+^ can act as hole traps. Recombination of electron–hole pairs at these trap states can result in the generation of Mn^2+^* and Yb^3+^* excited states, which undergo light emission. Figure 5(b) shows the steady-state emission spectra from Mn^2+^−Yb^3+^ co-doped CsPbCl_3_ NPs, in which optical excitation at 330 nm generates concerted perovskite NP centered emission at 410 nm, Mn^2+^ centered emission at 610 nm (^4^T_1_→^6^A_1_ transition), and Yb^3+^ centered emission at 980 nm (^2^F_5/2_→^2^F_7/2_ transition)—spanning the blue, orange-red, and NIR spectral regions. Figure 5(a) also predicts that Tb^3+^−Eu^3+^ is a viable co-dopant pair in CsPbCl_3_ NPs much like the ZnS:EuTb case (see Figure 3, *vide supra*); and characteristic Tb^3+^ and Eu^3+^ bands at 490, 545, 590, 616, 653, and 700 nm are observed from the time-gated emission spectrum of Tb^3+^−Eu^3+^ co-doped CsPbCl_3_ NPs [see Figure 5c].

Mn^2+^−Yb^3+^ and Tb^3+^−Eu^3+^ are not the only feasible co-dopant pairs in CsPbCl_3_ NPs. An examination of Figure 5(a) implies that Mn^2+^−Er^3+^ and Yb^3+^−Er^3+^ are likely emitter pairs and in fact explains the recent reports of Artizzu and co-workers^49,50^ where they observed Er^3+^ emission at 1550 nm (corresponding to the ^4^I_13/2_→^4^I_15/2_ transition) along with Mn^2+^ emissions in CsPbCl_3_:ErMn NPs and Er^3+^ and Yb^3+^ emissions in CsPbCl_3_:ErYb NPs. Interestingly, Artizzu and co-workers observed concerted Mn^2+^ and Nd^3+^ emissions in co-doped CsPbCl_3_ NPs.^50^ The underlying interdopant energy transfer mechanisms, including the role of inter-bandgap Mn (specifically the ^5^Mn^3+^ as discussed by De Angelis and co-workers) and Yb^2+^ levels to generate the Mn^2+^* and Yb^3+^*, symmetry effects, and spin selection rules, need further experimental exploration, however. Note that the placement of the Ln^3+^ levels below the valence band of the host does not affect the feasibility of them acting as trapping sites and finds ample literature precedents.^3,10,27,37^ The detailed nature of the trapping site and the coupling of the Ln^3+^ ions to the delocalized band states merit consideration. The Ln^3+^ doping percentage in these systems range from 1 – 9% and the density of states in the NPs is much lower than that of a bulk semiconductor. These facts imply that carrier trapping and detrapping rates can be much different from what one might imagine for dopant ion levels in a bulk semiconductor.

*The emission efficiencies of these Ln*^*3+*^*co-dopant pairs can be rationalized, and predicted, for other APbCl*_*3*_*perovskites with different A-site cation identities.*

## Future Perspective

In addition to its utility for creating f-f or d-f doped semiconductor NPs with bespoke emission properties, the semiempirical model for Ln dopant energy level positions in semiconductor NPs provides a platform for designing and performing more incisive experiments into the charge trapping mechanism of sensitization. Although the charge trapping model has proved consistent with a broad set of Ln-doped nanoparticle materials, the underlying charge localization/trapping and recombination processes that give rise to the lanthanide excited states have not been identified and probed directly. Time-resolved optical and extreme ultraviolet spectroscopic and kinetic studies of these materials is needed to identify the elementary steps in the sensitization mechanism. Coupled with these experimental studies, the need exists to improve the precision of computational approaches for determining the Ln^3+^ energy levels relative to semiconductor NP band edges.^52^ Such developments could provide the necessary quantitative understanding to predict material emission properties from first principles.

The charge trapping mechanism provides a framework for designing and improving luminophores to meet user-specific needs in applications and to affect charge flow in optoelectronic applications. For Ln^3+^ ions, the co-dopant energy alignments can be used to guide charge flow through dopant sites and to track the localization of the photogenerated charge carriers. These aspects are important considerations for device performance in light emitting diodes and in photovoltaics. Ln^3+^ emission brightening in NPs mediated by appropriate selection of co-dopants^53^ and excitation wavelengths^54^ have important implications for Ln^3+^ based imaging probes and theranostics. For example, we hypothesize that the manipulation of energy level alignments in doped NPs might prove useful in photodynamic therapy by tuning the interaction of charge carriers with reactive oxygen species.^55^ An organic – inorganic composite assembly can also be used to enhance Ln^3+^ emission by grafting a suitable polymer in doped NPs to act as a cosensitizer and surface ligand shell simultaneously.^56^ Band gap engineered Ln^3+^ doped NPs offer a wide range of applications in health and energy.^19−21^

A deep understanding of the charge trapping mechanism in doped nanoparticles could offer wholly new types of applications for doped NPs in chemistry. For example, imbedding Ln^3+^ dopants in photocatalysts could be used to report on the redox state of active sites and their energy. Alternatively, co-doped NPs with informed knowledge of their relative energetics could be used to introduce inter-bandgap states that promote charge separation of photogenerated electron–hole pairs. The charge trapping mechanism provides a new perspective on carrier kinetics and electronic energy flow in nanoparticles with important implications.
